# Supplementary material for: Development and Characterization of a Novel Peptide—Drug Conjugate with DM1 for Treatment of FGFR2-Positive Tumors
Source: Biomedicines. 2021 Jul 21;9(8):849. doi: 10.3390/biomedicines9080849 (PMC8389697; doi:10.3390/biomedicines9080849)
Supplement: Supplementary file 1 [file biomedicines-09-00849-s001.zip › biomedicines-1271044-supplementary.pdf]

Supplementary data

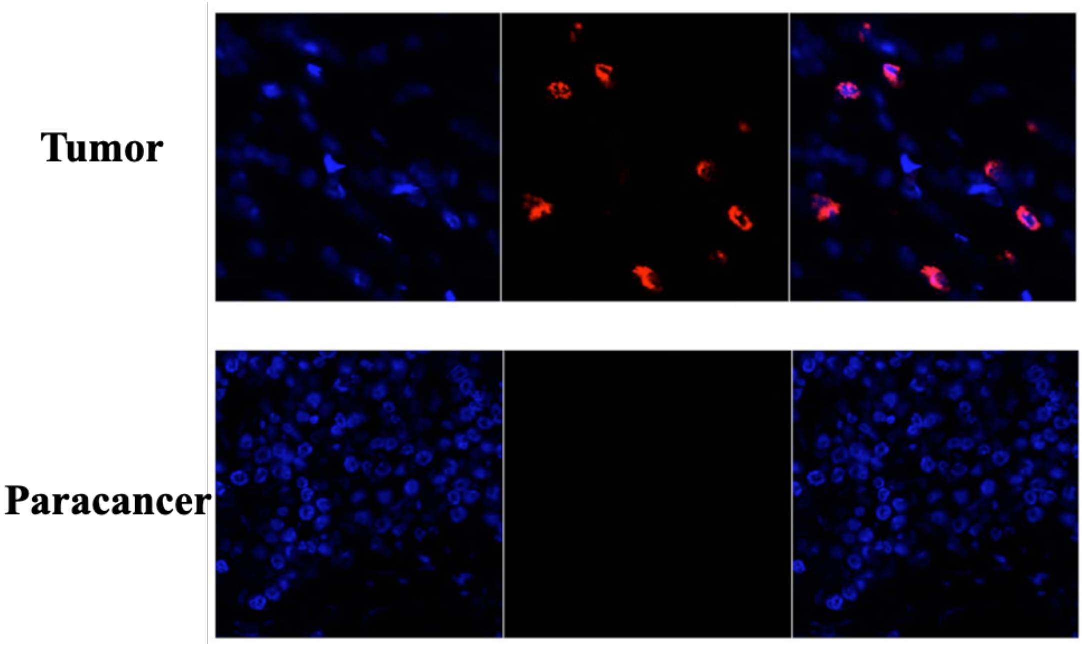

Figure S1. The observation of fluorescence signal of LLC2B-L-Cy5.5 from human esophagus cancer and paracancer of esophagus cancer by confocal microscope.

List of amino acids of 5'-XXXQ'A'E'XX-3' OBOC library:

X: 42 natural/non-natural amino acids

Amino acids at position Q': Q/Hocit/n/Cit

Amino acids at position A': A/G/Aib/V/Acpc

Amino acids at position E': E/d/Aad

Table S1 42 Fmoc-amino Acids

#1<br>Fmoc- Orn(Boc)-OH<br>MW: 454.5 (132.12)<br>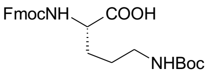 	#2<br>Fmoc-Acpc-OH<br>MW: 323.35 (101.1)<br>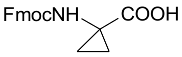 	#3<br>Fmoc-L-HoCit<br>MW: 411.4<br>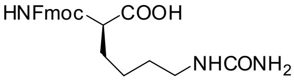 	#4<br>Fmoc-Hyp(tBu)-OH<br>MW: 409.0<br>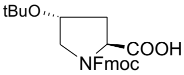
#5<br>Fmoc-Aad(OtBu)-OH<br>MW: 439.6<br>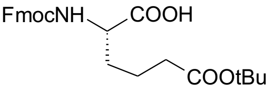 	#6<br>Fmoc-D-3-Pal-OH<br>MW: 388.4<br>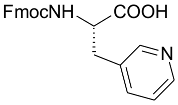 	#7<br>Fmoc- Aib-OH<br>MW: 325.5<br>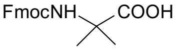 	#8<br>Fmoc- Nva-OH<br>MW: 339.4<br>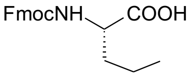
#9<br>Fmoc-Dpr(Boc)-OH<br>MW: 426.4<br>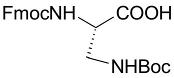 	#10<br>Fmoc-D-Tyr(Me)-OH<br>MW: 417.47<br>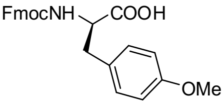 	#11<br>Fmoc-L-Phg-OH<br>MW: 373.4<br>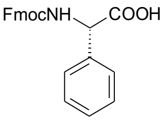 	#12<br>Fmoc-Nle-OH<br>MW: 353.4<br>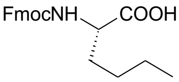
#13<br>N-Fmoc-amino-(4-N-Boc-piperidinyl) carboxylic acid<br>MW:466.53 (4-Apc)	#14<br>Fmoc-Phe(4-Me)-OH<br>MW: 401.46<br>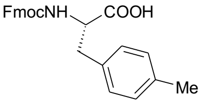 	#15<br>Fmoc-Aic-OH<br>MW: 399.45<br>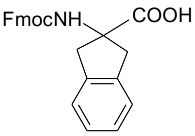 	#16<br>Fmoc-D-Phe(3-Cl)-OH<br>MW: 421.9<br>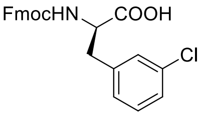

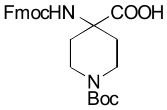			
#17<br>Fmoc-D-HoPhe-OH<br>MW: 401.5<br>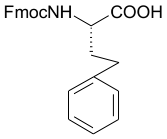	#18<br>Fmoc-D-Chg-OH<br>MW: 379.4<br>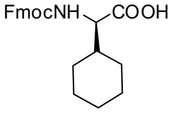	#19<br>Fmoc-Bpa-OH<br>MW: 491.5<br>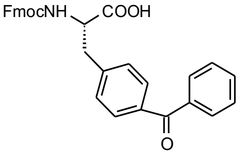	#20<br>Fmoc-Arg(Pbf)-OH<br>MW: 648.8 R<br>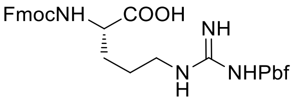
#21<br>Fmoc-D-2-Nal-OH<br>MW: 437.47<br>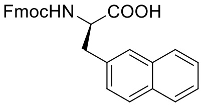	#22<br>Fmoc-L-1-Nal-OH<br>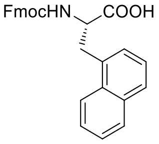	#23<br>Fmoc-Phe(3,4-diCl)-OH<br>MW: 456.4<br>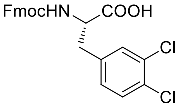	#24<br>Fmpc-Cha-OH<br>MW: 393.48<br>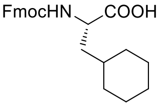
#25<br>Fmoc-D-Ala-OH<br>MW: 311.3 a<br>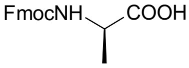	#26<br>Fmoc-D-Glu(OtBu)-OH<br>MW: 425.5 e<br>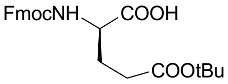	#27<br>Fmoc-D-Asn(Trt)-OH<br>MW: 596.7 n<br>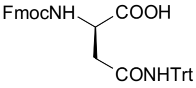	#28<br>Fmoc-Ile-OH<br>MW:353.4 I<br>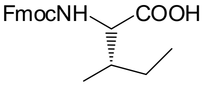
#29<br>Fmoc-D-Leu-OH<br>MW: 353.4 l<br>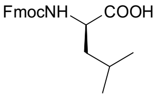	#30<br>Fmoc-D-Lys(Boc)-OH<br>MW:468.6 k<br>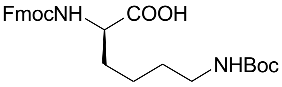	#31<br>Fmoc-D-Ser(tBu)-OH<br>MW: 383.4 s<br>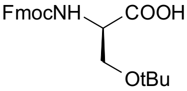	#32<br>Fmoc-D-Met-OH<br>MW: 371.5 m<br>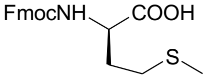
#33<br>Fmoc-D-Phe-OH<br>MW: 387.4 f<br>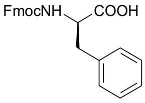	#34<br>Fmoc-D-Pro-OH<br>MW: 337.4 p<br>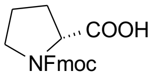	#35<br>Fmoc-L-Thr(tBu)-OH<br>MW: 397.5 T<br>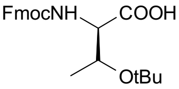	#36<br>Fmoc-L-Val-OH<br>MW: 339.4 V<br>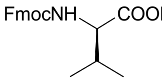

#37<br>Fmoc-D-Trp(Boc)-OH<br>MW: 526.6 w<br>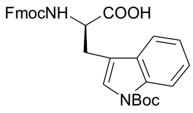	#38<br>Fmoc-D-His(Trt)-OH<br>MW: 619.7 h<br>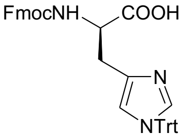	#39<br>Fmoc-Asp(OtBu)-OH<br>MW: 411.5 D<br>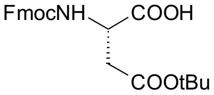	#40<br>Fmoc-Gln(Trt)-OH<br>MW: 610.7<br>Q<br>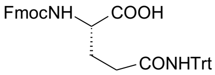
#41<br>Fmoc-Tyr(tBu)-OH<br>MW: 459.6 Y<br>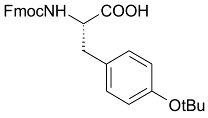	#42<br>Fmoc-Gly-OH<br>MW: 297.3 G		

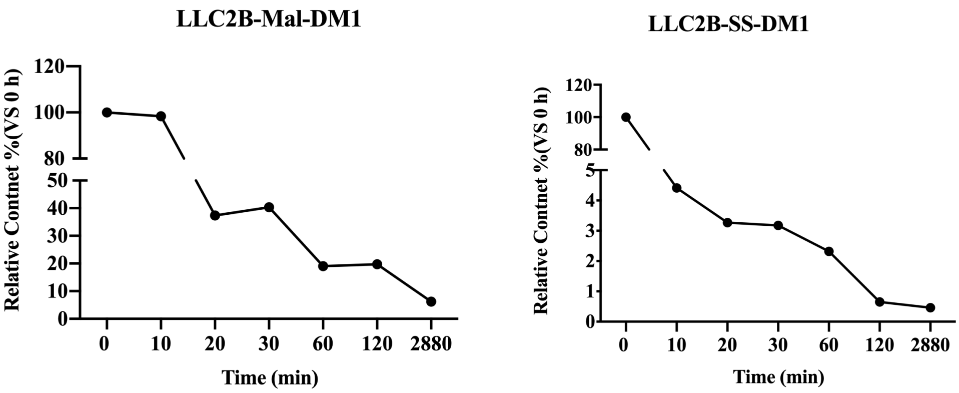

Figure S2. The remaining percentage of LLC2B-Mal-DM1, LLC2B-SS-DM1 that's being mixed with murine plasma at different time points

Table S2 Tumor volume (mm^3) in the PBS, free DM1, LLC2B-SS-DM1group

	PBS	0.5 mg Free DM1/kg	0.5 mg DM1 equiv./kg	1.0 mg DM1 equiv./kg	2.0 mg DM1 equiv./kg
**0 d**	156.87±67.61	175.68±59.92	101.07±81.57	118.88±32.94	126.80±38.96
**2 d**	679.76±202.24	314.80±122.30	226.16±129.38	184.22±33.22	223.67±53.70
**4 d**	876.51±185.09	551.00±238.38	304.04±157.48	283.28±88.46	321.17±107.85
**6 d**	944.05±289.43	557.43±209.97	389.20±198.90	272.22±113.49	280.78±95.68
**8 d**	1203.71±398.07	654.39±219.50	426.44±198.04	334.59±117.92	360.58±82.08
**10 d**	1290.61±441.17	650.06±336.33	499.04±194.24	395.08±132.16	273.01±107.62
**12 d**	1529.18±536.95	772.79±324.13	541.61±312.25	388.28±121.27	355.51±151.42
**14 d**	1707.01±431.41	788.24±379.02	593.61±364.12	393.89±187.58	461.17±136.73
**16 d**	1895.95±561.93	873.66±422.26	781.68±522.51	466.38±156.74	411.41±168.66
**18 d**	1977.21±609.36	893.32±477.86	790.28±366.88	564.61±188.93	540.99±193.86
**20 d**	2315.02±430.85	1110.11±531.65	713.09±359.65	567.38±204.08	604.21±201.81

Table S3 Tumor volume (mm^3) in the PBS, free DM1, LLC2B-Mal-DM1group

	PBS	1.0 mg Free DM1/kg	1.0 mg DM1 equiv./kg	2.0 mg DM1 equiv./kg	4.0 mg DM1 equiv./kg
**0 d**	212.98±113.45	167.91±51.37	123.92±35.19	204.16±84.38	187.26±94.71
**2 d**	651.09±182.05	333.54±106.62	184.85±129.03	329.89±146.35	408.83±131.11
**4 d**	880.35±160.43	499.47±119.89	301.27±140.11	461.08±181.64	535.64±174.77
**6 d**	989.95±262.97	653.81±266.46	357.78±258.20	651.88±257.36	706.23±296.92
**8 d**	1223.14±346.38	985.99±232.74	488.31±344.39	678.53±157.79	754.99±348.08
**10 d**	1312.66±383.96	838.17±157.44	562.06±270.65	552.08±177.84	858.62±298.09
**12 d**	1539.65±465.37	1176.08±97.85	705.13±200.23	787.04±251.09	806.65±165.51
**14 d**	1662.22±381.58	1287.25±178.41	752.16±212.63	817.69±244.31	1050.39±437.35
**16 d**	1898.21±486.66	1286.39±189.90	773.25±228.24	890.28±412.67	965.55±308.55
**18 d**	1992.15±528.35	1395.67±243.57	904.65±254.13	1025.35±439.18	1047.92±374.01
**20 d**	2349.32±377.83	1727.06±280.01	1250.63±342.99	1130.29±354.15	1166.16±373.54
